# Adenosine A2A receptor signaling in neuroinflammation, glial modulation, and mechanisms associated with chronic pain

**DOI:** 10.1007/s11302-026-10167-1

**Published:** 2026-06-10

**Authors:** Clara Andressa Barros, Jéssica Gonçalves Rangel, Bruno de Oliveira Cruz, Leonardo Pereira De Araújo, Giovane Galdino

**Affiliations:** 1 Laboratory of Physiological Sciences, Department of Biophysics and Physiology, Federal University of Piauí, UFPI-PI), Teresina, Piauí Brazil; 2 Pharmaceutical Sciences, Federal University of Alfenas (UNIFAL-MG), Alfenas, Minas Gerais Brazil; 3 Laboratory of Molecular Biology of Insects, Federal University of Alfenas (UNIFAL-MG), Alfenas, Minas Gerais Brazil; 4 Laboratory of Molecular Biology of Microorganisms, Federal University of Alfenas (UNIFAL-MG), Alfenas, Minas Gerais Brazil; 5 Pain Neuroimmunobiology Laboratory, Center for Experimental Biology, Federal University of Alfenas (UNIFAL-MG), Jovino Ferandes Sales St. 2600, Alfenas, Minas Gerais 70037130-001 Brazil

**Keywords:** Adenosine A2A receptor, Purinergic signaling, Chronic pain, Neuroinflammation, Glial activation

## Abstract

Chronic pain is increasingly recognized as a complex and multidimensional condition that extends beyond peripheral nociceptive input, encompassing maladaptive central mechanisms such as persistent neuroinflammation, glial dysfunction, aberrant synaptic plasticity, and disturbances in affective–motivational circuits. Adenosine, an endogenous purine nucleoside, plays a pivotal role in central nervous system homeostasis through activation of four G protein–coupled receptors (A1, A2A, A2B, and A3). Among these, the adenosine A2A receptor (A2AR) has emerged as an important modulator of neuroimmune signaling, astrocyte–neuron communication, synaptic excitability, and behavior. Although adenosinergic signaling has been extensively investigated in the context of pain and neurological disorders, the specific contribution of A2AR to pain-related mechanisms is often addressed indirectly, as many studies focus on upstream neurobiological processes rather than nociception itself. In this narrative review, we integrate recent experimental, translational and observational evidence from in silico, in vitro, ex vivo, in vivo, and human studies to examine how A2AR-dependent signaling may influence mechanisms associated with pain modulation. The literature is organized according to conceptual and mechanistic criteria into four thematic axes: (i) neuroinflammation and central sensitization, (ii) astrocytic regulation and synaptic modulation, (iii) neurological disorders with pain-related components, and (iv) affective and emotional processes associated with the pain experience. Across these domains, convergent evidence suggests that A2AR signaling is involved in interactions between glial activation, inflammatory signaling, synaptic plasticity, and affective regulation. Together, these findings provide a framework for understanding how A2AR-related mechanisms contribute to process associated with chronic pain development and persistence, even in experimental contexts not explicitly designed to assess nociception. This integrative perspective supports further investigation of A2AR as a potential therapeutic target in chronic pain and related neuroinflammatory conditions.

## Introduction

Adenosine is an endogenous purine nucleoside that is ubiquitous yet short-lived, acting as a central regulator of neuromodulation, immune homeostasis, and metabolic balance [1–3]. In the extracellular space, adenosine signals through four G protein–coupled receptors—A1, A2A, A2B, and A3—expressed across the central nervous system (CNS), glial cells, vascular endothelium, and peripheral immune compartments [2, 3]. By coupling metabolic status to cellular excitability and immune tone, adenosinergic signaling shapes synaptic transmission, neuronal responsiveness, and inflammatory programs under both physiological and pathological conditions [1–3].

Among adenosine receptors, the adenosine A2A receptor (A2AR) has attracted particular attention due to its prominent role in orchestrating complex neurobiological processes [4–6]. Canonically coupled to Gs/Golf proteins, A2AR activation increases intracellular cAMP and engages downstream signaling cascades involving protein kinase A (PKA), cAMP response element–binding protein (CREB), and extracellular signal–regulated kinases 1/2 (ERK1/2). In parallel, A2AR signaling can recruit β-arrestin–dependent mechanisms that influence receptor internalization and pathway bias [4–6]. A2ARs are broadly distributed in the CNS and are expressed in pre- and postsynaptic neurons, astrocytes, microglia, and vascular cells, with high density in the striatum and nucleus accumbens, but also in regions directly relevant to sensory processing and pain modulation, including the hippocampus and spinal cord—particularly the substantia gelatinosa of lamina II [1–3, 6].

Functionally, A2AR signaling regulates glial reactivity, astrocyte–neuron communication, neurotransmitter release, and neuroinflammatory responses [1–3, 6, 7]. Importantly, experimental evidence indicates that A2AR activation may yield divergent outcomes depending on cellular context, anatomical compartment, and disease stage: in some settings A2AR signaling appears neuroprotective, whereas in others it promotes maladaptive plasticity and persistent inflammation. This context dependence has positioned A2AR as a therapeutically relevant target across multiple neurological disorders, including Parkinson’s disease, Alzheimer’s disease, Huntington’s disease, cerebral ischemia, and spinal cord injury [3, 7–9].

In parallel, the field increasingly recognizes that microglial and astrocytic activation, sustained release of inflammatory mediators, excitatory–inhibitory imbalance, and dysfunction of affective and sleep-related circuits constitute shared central mechanisms across a wide range of neurological and psychiatric disorders [1–6]. These same processes are also frequently implicated in chronic pain mechanisms, particularly through central sensitization, in which nociceptive processing becomes amplified and persistently dysregulated [2, 3].

Consistent with this framework, studies in neuropathic and inflammatory pain models suggest that adenosine signaling modulates neuronal and non-neuronal function, shapes primary afferent transmission, and influences pain-related behaviors [1, 3, 6, 8, 10]. However, the role of A2AR remains debated, with reports supporting both pro- and antinociceptive actions depending on the site of action, cell type, and inflammatory milieu [1–3, 11]. Against this backdrop, the present review compiles and critically organizes recent experimental and observational evidence linking A2AR to neuroinflammation, glial regulation, synaptic modulation, and affective behavior. Notably, the evidence discussed spans different levels of association with chronic pain. While some studies directly assess nociceptive outcomes in established pain models, a substantial portion of the literature focuses on upstream neurobiological processes, such as glial activation, inflammatory signaling, and synaptic plasticity, that are frequently implicated in mechanisms of pain modulation but are not always evaluated in terms of pain-related behavior.

Accordingly, this review aims to distinguish between direct and indirect lines of evidence and to highlight convergent pathways through which A2AR signaling may influence mechanisms involved in pain processing and modulation. By adopting this integrative and critically contextualized perspective, we seek to more precisely define the extent to which A2AR-dependent processes can be interpreted within the framework of chronic pain, while acknowledging current limitations and areas requiring further investigation.

## Search strategy and selection criteria

A targeted search was conducted in PubMed to identify original studies addressing the role of the adenosine A2A receptor (A2AR/ADORA2A) in neuroimmune pathways relevant to neuropathic pain and related central mechanisms. The search query combined receptor terms with injury models and neuroimmune keywords:

("A2A receptor" OR ADORA2A OR "adenosine A2A") AND (CCI OR "chronic constriction injury" OR SNI OR "spared nerve injury" OR "spinal cord injury" OR SCI) AND (microglia OR astrocytes OR neuroinflammation).

To strengthen the interpretation of A2AR-related mechanisms within the context of chronic pain, additional targeted searches were performed to identify primary studies directly assessing nociceptive outcomes in neuropathic and inflammatory pain models. These complementary searches were not restricted to the initial query string and were guided by key mechanistic themes identified during data extraction (e.g., microglial activation, cytokine signaling, and synaptic plasticity). Studies identified through this approach were incorporated to provide direct evidence linking specific neurobiological processes to pain-related outcomes, thereby supporting the distinction between direct and indirect lines of evidence adopted in this review.

Only studies published within the last ten years were considered. Reviews, editorials, conference abstracts, and non-original publications were excluded. After screening, 20 original studies were retained. In addition to studies conducted in established neuropathic pain models, relevant experimental work investigating A2AR-dependent neuroimmune and neurobiological mechanisms was also considered when these processes are widely implicated in pain modulation, even if nociceptive outcomes were not directly assessed.

For each study, data extraction included publication metadata (authors, year, PMID), experimental design (in vivo, in vitro, ex vivo, in silico, or human observational), injury model (CCI, SNI, SCI), A2AR manipulation (agonists, antagonists, or genetic approaches), primary cellular targets (microglia, astrocytes, neuron–glia interactions), neuroimmune outcomes (cytokines, glial markers, signaling pathways), and, when available, behavioral or physiological correlates relevant to neuropathic pain (e.g., allodynia, hyperalgesia, functional outcomes). The resulting dataset was used to identify convergent mechanistic patterns and to distinguish between direct and indirect lines of evidence linking A2AR signaling to central processes involved in pain.

## Characterization of the included studies and methodological approach

Using the predefined strategy, 37 records were initially retrieved. After restricting to recent publications and excluding review articles, 20 original studies were included. The final set encompasses in silico, in vitro, ex vivo, in vivo, and human observational designs, capturing the breadth of adenosinergic and A2AR-related mechanisms across cellular, circuit-level, and translational contexts within the CNS.

Because the objective of this review was mechanistic integration rather than formal evidence synthesis, studies were not organized under a PRISMA-based systematic framework. Instead, articles were grouped according to conceptual and mechanistic criteria: (i) the experimental model employed, (ii) involvement of A2AR in glial and/or neuroinflammatory processes, and (iii) relevance to biological mechanisms associated with central sensitization and pain modulation. Accordingly, the literature was organized into four thematic axes: (1) neuroinflammation and central sensitization; (2) astroglial signaling and synaptic modulation; (3) neurological disorders with potential relevance to pain-relevant components; and (4) affective and emotional regulation associated with pain.

Although pain was not the primary outcome in all studies, their inclusion was based on the relevance of the investigated mechanisms to processes commonly implicated in pain modulation. Chronic pain is increasingly understood as a multifactorial condition involving neuroinflammatory signaling, glial activation, synaptic plasticity, and alterations in affective and regulatory circuits. However, not all such disturbances necessarily result in chronic pain, and their contribution may depend on context, severity, and system-level interactions.

Therefore, studies focused on neurodegenerative disorders, mood alterations, sleep-related phenotypes, retinal degeneration, demyelination, or brain injury were included insofar as they provide insight into biological mechanisms that may be relevant to pain-related processes, even when nociceptive outcomes were not directly assessed. Table 1 summarizes the main characteristics of the included studies, including their experimental context and relevance to pain-related mechanisms.

**Table 1:** Summary of the studies included in this review addressing adenosine A2A receptor signaling across experimental models relevant to neuroinflammation, glial dysfunction, synaptic modulation, and pain-related processes

Topics	Author, year, country and DOI	Experimental model	Main findings	Pain-related translational significance
A2A receptor signaling in neuroinflammatory and glial mechanisms relevant to pain biology	Duan et al., 2018, China; 10.1038/s41598-018–25031-5	In vitro BV2 microglial cell line exposed to hypoxia and low glucose, with adenosine A2A receptor (A2AR) activation (CGS21680) or inactivation (SCH58261) and CF (cystatin F) knockdown to probe mechanisms of neuroinflammation	Activation of A2A receptors increased cystatin F expression and enhanced pro-inflammatory cytokine (IL-1β, IL-6, TNF-α) production in hypoxic microglia, whereas A2AR inactivation (SCH58261) reduced CF expression and attenuated cytokine increases. The results indicate that A2AR-mediated neuroinflammation involves PKA/CREB and PKC/CREB or ERK1/2 signaling pathways, and that CF contributes to A2AR-driven inflammatory responses	Increased production of pro-inflammatory cytokines may indirectly contribute to chronic pain development, as IL-1β, IL-6, and TNF-α are established mediators of neuroinflammation and central sensitization
	Madeira et al., 2023, Portugal; 10.1007/s00018-023–04983-6	Mouse models of Alzheimer’s disease (APP/PS1 transgenic mice and Aβ₁₋₄₂–treated hippocampal slices) examining astrocytic Connexin 43 (Cx43) hemichannel activity	Adenosine A2A receptor (A2AR) blockade (pharmacological or genetic) prevented Aβ-induced dysregulation of astrocytic Cx43 hemichannel activity in hippocampal slices, showing that A2AR modulates astrocytic hemichannel dysfunction in Alzheimer’s models	Astrocytic hemichannel dysregulation may alter gliotransmission and extracellular signaling, mechanisms potentially involved in maladaptive synaptic plasticity associated with chronic pain
	Fouda et al., 2020, USA; 10.1016/j.lfs.2020.117598	Diabetes mellitus (DM) rat model with groups stratified by sex and estrogen status (male, sham-operated female, ovariectomized female, ovariectomized with estrogen supplementation); cardiac autonomic function and hypothalamic neuroinflammation assessed	In female diabetic rats with estrogen (E₂), there was autonomic dysregulation (sympathetic dominance) accompanied by hypothalamic increases in proinflammatory markers and upregulation of adenosine receptors, including A2A receptors (A2AR), suggesting that estrogen-dependent upregulation of proinflammatory A1 and A2A receptors in the paraventricular nucleus contributes to heightened neuroinflammation and cardiac autonomic dysfunction	Hypothalamic neuroinflammation and autonomic dysfunction may indirectly influence pain-related neuroimmune responses under chronic metabolic stress conditions
	Marciante et al., 2023, USA; 10.1152/jn.00035.2023	Adult Sprague–Dawley rats treated with low-dose LPS to induce mild inflammation, combined with moderate acute intermittent hypoxia (mAIH) to elicit phrenic long-term facilitation (pLTF) in vivo	Mild inflammation increased spinal adenosine levels and A2A receptor activation in the cervical spinal cord, which abolished mAIH-induced phrenic long-term facilitation (pLTF); inhibition of A2A receptors (intrathecal MSX-3) rescued pLTF, demonstrating that spinal A2AR activation mediates inflammation-related impairment of respiratory motor plasticity	Inflammation-induced impairment of adaptive neural plasticity suggests that excessive A2AR activation may contribute to maladaptive plasticity mechanisms relevant to chronic pain sensitization
	Jo et al., 2023, South Korea; 10.1038/s41598-023–32744-9	In vivo ischemia/inflammation models (animal models) treated with polydeoxyribonucleotide (PDRN) as a selective A2A receptor agonist	Activation of adenosine A2A receptor (ADORA2A/A2AR) by PDRN modulated inflammation and promoted tissue repair in ischemic/inflammatory conditions, showing the therapeutic benefit of A2AR activation in reducing inflammation/ischemia-related injury	Modulation of inflammatory and ischemic injury pathways may influence neuroimmune mechanisms that contribute indirectly to pain generation in inflammatory conditions
Astrocytic A2ARs in synaptic regulation and glia–neuron communication	Amato et al., 2022, Italy; 10.3390/ijms23042326	In vitro experiments using primary astrocyte cultures isolated from adult rat striatum, combined with pharmacological manipulation of purinergic and oxytocin receptors, calcium imaging and glutamate release assays	Adenosine A2A receptors (A2AR) form a functional heterodimer with oxytocin receptors (OTR) in adult striatal astrocytes, and A2AR activation negatively modulates OTR-mediated signaling, leading to reduced astrocytic Ca^2^⁺ responses and glutamate release, identifying A2AR as a key inhibitory regulator of astrocyte-dependent glutamatergic modulation	Regulation of astrocytic Ca^2^⁺ signaling and glutamate release may influence excitatory neurotransmission and network excitability relevant to pain sensitization
	Pelassa et al., 2019, Italy; 10.3390/ijms20102457	In vitro primary adult rat striatal astrocytes and striatal slices, analyzed with biochemical and biophysical assays to investigate receptor interactions	Adenosine A2A receptors (A2AR) are co-expressed with dopamine D2 receptors (D2R) on adult rat striatal astrocytes, and A2AR–D2R heteromerization was directly demonstrated at the astrocyte plasma membrane; these A2AR–D2R heteromers regulate glutamate release from astrocyte processes, revealing a receptor-receptor interaction mechanism that controls striatal glutamatergic transmission	Regulation of astrocytic Ca^2^⁺ signaling and glutamate release may influence excitatory neurotransmission and network excitability relevant to pain sensitization
	Amato et al., 2024, Italy; 10.3390/ijms25168610	In vitro study of striatal astrocytes from adult rodents (biochemical, imaging and functional assays on astrocytic processes)	Evidence for a high-order receptor complex containing adenosine A2A receptor (A2AR), dopamine D2 receptor and oxytocin receptor (OTR) in striatal astrocytes; A2AR activation inhibited D2-mediated signaling and its facilitation by OTR in controlling intracellular Ca^2^⁺ signaling and glutamate release, indicating an inhibitory modulatory role of A2AR within the A2A-D2-OTR complex	Fine regulation of astrocytic glutamatergic signaling may contribute to mechanisms controlling neural excitability and maladaptive synaptic plasticity linked to chronic pain
	Dias et al., 2022, Portugal; 10.1007/s00018-022–04492-y	In vitro primary cultured rat astrocytes exposed to Aβ1–42 peptides to model Alzheimer’s-related astrocyte dysfunction and assay purinergic receptor signaling	Exposure to Aβ1–42 disrupted adenosine A2A receptor (A2AR) regulation of ATP-evoked astrocytic calcium responses and the interplay between P2X7 and P2Y1 purinergic receptors; blockade of A2AR with SCH-58261 modulated these Ca^2^⁺ responses and prevented some receptor changes induced by Aβ, indicating that A2AR control of astrocytic Ca^2^⁺ dynamics is impaired by Aβ1–42	Disrupted astrocytic calcium signaling may impair glial homeostasis and neurotransmission balance, processes potentially associated with persistent pain states
	Queiroga et al., 2016, Portugal; 10.1242/jcs.187260	In vitro astrocyte–neuron co-culture system in mice, where astrocytes were pre-treated with carbon monoxide (CO) and then co-cultured with neurons to assess neuroprotection mechanisms, including purinergic signaling components	CO-treated astrocytes reduced neuronal cell death via paracrine purinergic signaling; antagonists of P1 adenosine receptors and knockdown of neuronal A2A receptor (A2AR; Adora2a) expression reversed the neuroprotective effect, indicating that A2AR is required for CO-induced astrocyte-mediated neuroprotection	A2AR-dependent astrocyte–neuron communication may influence neuronal survival and synaptic regulation, which may indirectly affect pain-related circuit integrity
A2A Receptor in neurological disorders with pain components	Casati et al., 2016, Italy; 10.1016/j.jns.2015.12.040	Human observational case–control study analyzing peripheral blood mononuclear cells (PBMCs) from idiopathic normal-pressure hydrocephalus (iNPH) patients and age-matched healthy controls, with qPCR and Western blot assessment of adenosine A1 and A2A receptor gene and protein expression	A2A receptor (A2AR) mRNA expression was significantly down-regulated in PBMCs from iNPH patients compared to controls, with a trend toward reduced A2AR protein levels, indicating a systemic alteration of the adenosinergic system in iNPH and suggesting that reduced A2AR signaling may be associated with vascular and immune dysregulation in the disease	Systemic alterations in A2AR expression may reflect broader neuroimmune dysregulation associated with neurological disorders that can present pain-related manifestations
	Zhao et al., 2017, China; 10.1038/s41598-017–02505-6	In vivo rodent traumatic brain injury (TBI) model, with assessment of adenosine A2A receptor (A2AR) inactivation effects on cerebral water channel regulation and injury outcomes	After TBI, perivascular dysregulation of aquaporin-4 (AQP4) in the hippocampal CA1 region was observed; inactivation of A2A receptors alleviated this dysregulation and was associated with improved injury markers, indicating that A2AR blockade mitigates TBI-induced AQP4 polarity disruption and may protect against interstitial solute accumulation post-injury	Preservation of glymphatic and astrocytic homeostasis following A2AR blockade may reduce neuronal dysfunction that could indirectly affect sensory processing after injury
	Akbari et al., 2018, Iran; 10.1016/j.lfs.2018.05.007	In vivo LPC-induced focal demyelination in rat hippocampal fimbria, treated with intracerebroventricular A2A receptor antagonist (SCH58261), with spatial memory (Morris water maze) and myelination/glial activation assessed post-lesion	Blockade of adenosine A2A receptors with SCH58261 prevented spatial memory impairment, reduced the extent of LPC-induced demyelination, and attenuated astrocyte and microglia activation in the fimbria, demonstrating that A2AR inactivation exerts pro-myelinating, anti-inflammatory and cognitive-protective effects in a focal demyelination context, supporting A2AR blockade as a potential therapeutic strategy for multiple sclerosis	Reduced demyelination and glial activation may preserve axonal integrity, potentially limiting mechanisms involved in neuropathic pain development
	Madeira et al., 2018, Portugal; 10.1038/s41598-018–20733-2	In vitro study using human immortalized microglial cells and ARPE-19 retinal pigment epithelial cells exposed to inflammatory stimuli, with and without selective adenosine A2A receptor (A2AR) antagonist SCH58261	Blockade of A2AR with SCH58261 in microglia reduced expression of pro-inflammatory mediators, attenuated complement and inflammasome activation in both microglia and ARPE-19 cells exposed to microglia-conditioned medium, and enhanced clearance of apoptotic photoreceptors, indicating that A2AR antagonism mitigates microglia-mediated inflammation and protects retinal cells in an AMD-related in vitro model	Attenuation of microglia-mediated inflammation may reduce inflammatory signaling pathways associated with pain chronification
	Aires et al., 2019, Portugal; 10.1038/s41598-019–53627-y	In vivo type 1 diabetic mouse model (C57BL/6 J mice) induced with streptozotocin; intravitreal injections of an adenosine A2A receptor (A2AR) antagonist given weekly for 4 weeks	Intravitreal administration of an A2AR antagonist suppressed microglia reactivity and neuroinflammation in the diabetic retina, reduced retinal vascular leakage, attenuated retinal thinning, decreased retinal cell death, and protected retinal ganglion cells in diabetic mice, demonstrating that A2AR blockade confers retinal protection and limits diabetes-associated retinal degeneration	Attenuation of microglia-mediated inflammation may reduce inflammatory signaling pathways associated with pain chronification
Affective dimension of pain: Involvement of the A2A receptor in behavior, mood, and pain perception	Scharbarg et al., 2016, France; 10.1038/srep19107	Ex vivo mouse brain slice preparations of the ventrolateral preoptic nucleus (VLPO) were used to study how glucose influences local adenosine release and neuronal responses, including electrophysiology and purine biosensor measurements	Astrocyte-derived adenosine increased in response to glucose and wakefulness, and activation by an A2A receptor agonist (CGS-21680) depolarized sleep-promoting VLPO neurons, demonstrating that A2A receptor signaling contributes to adenosine’s modulation of sleep-related neuronal activity, linking metabolic cues to sleep–wake control	A2AR-mediated modulation of sleep-related neuronal activity may influence sleep disturbances commonly associated with chronic pain conditions
	Jonsson & Eysteinsson, 2017, Iceland; 10.1167/iovs.15–18,024	In vivo Sprague Dawley rats, with intravitreal injection of adenosine receptor agonists and antagonists to assess the role of retinal A2A and A3 adenosine receptors in the electroretinogram (ERG) responses	Retinal A2A adenosine receptor (A2AR) activation with a selective agonist (CGS21680) reduced the ERG b-wave amplitude and oscillatory potentials, while blockade with an A2AR antagonist (ZM241385) also decreased the scotopic b-wave, indicating that A2AR modulates retinal electrophysiological responses and contributes to the generation of specific ERG components in the rat retina	A2AR regulation of retinal sensory processing supports a broader role for adenosinergic signaling in modulation of sensory system excitability
	Nguyen et al., 2022, South Korea; 10.1016/j.jad.2022.09.108	In silico toxicogenomic and network-based analysis integrating CTD, GeneMANIA, Metascape, MIENTURNET and Cytoscape to identify curcumin-targeted genes, transcription factors and miRNAs associated with depression	Curcumin was identified as targeting ADORA2A (A2AR), with predicted downregulation of ADORA2A-associated miRNA expression, supporting an A2AR-linked antidepressant mechanism consistent with reduced adenosinergic signaling involved in depressive phenotypes	Predicted A2AR involvement in antidepressant-associated pathways suggests potential relevance to affective disturbances frequently comorbid with chronic pain
	Zhao et al., 2024, China; 10.1002/advs.202404188	Transgenic mice with brain-specific overexpression of RagA	RagA overexpression increased ADORA2A (A2AR) expression in the prefrontal cortex, and pharmacological blockade of A2AR with istradefylline alleviated depressive-like behavior, demonstrating a functional role of the RagA–p70S6K–A2AR signaling axis	Improvement of depressive-like behavior following A2AR blockade suggests a role for A2AR signaling in affective processes relevant to pain perception
	Zaki et al., 2025, Canada; 10.3390/ijms26125680	Male Sprague–Dawley rats subjected to pial vessel disruption (PVD) to model small-vessel stroke; treated with the A2A receptor antagonist istradefylline or vehicle	Selective blockade of adenosine A2A receptors (A2AR) with istradefylline attenuated stroke-induced anxiety/depressive-like behaviors, improved cognitive and motor outcomes, and reduced neurodegeneration and neuroinflammation, linking A2AR antagonism to neuroprotection in cerebral ischemia	Improvement of depressive-like behavior following A2AR blockade suggests a role for A2AR signaling in affective processes relevant to pain perception

## A2A receptor signaling in neuroinflammatory and glial mechanisms relevant to pain biology

Experimental studies across multiple cellular and animal models indicate that A2AR signaling participates in the regulation of neuroinflammatory and glial responses under conditions of inflammatory, metabolic, or neurodegenerative stress [12–26]. In these contexts, A2AR activation has been associated with modulation of cytokine production, astrocytic and microglial reactivity, and alterations in neural plasticity-related processes [12–29]. Conversely, pharmacological or genetic modulation of A2AR-associated pathways has been shown to attenuate inflammatory signaling and partially restore impaired functional outcomes in selected experimental paradigms [10, 22–26, 30–38]. Collectively, these findings support a broader role for A2AR in regulating neuroimmune and neurobiological mechanisms that may be relevant to central nervous system dysfunction across pathological conditions.

### Microglial A2AR signaling and pro-inflammatory cytokine production

Experimental evidence indicates that A2AR signaling modulates inflammatory responses in microglial cells under pathological stress conditions. Activated microglia have been shown to upregulate A2AR expression, and receptor stimulation may influence the release of inflammatory mediators depending on the cellular context [12]. In BV2 microglial cells exposed to hypoxia and low-glucose environments, pharmacological activation of A2AR increased expression of cystatin F (CF), a protein associated with activated microglial phenotypes, while also promoting the production of pro-inflammatory cytokines including IL-1β, IL-6, and TNF-α [13]. Mechanistically, this response involved activation of PKA/CREB-, PKC/CREB-, and ERK1/2-dependent signaling pathways, suggesting that A2AR stimulation contributes to intracellular signaling programs associated with inflammatory amplification [13]. Notably, silencing of CF attenuated cytokine induction, supporting the existence of an A2AR–CF signaling axis involved in regulating microglial inflammatory output [13].

Supporting these findings, studies in neonatal rat microglia demonstrated that pharmacological A2AR activation partially increased expression of pro-inflammatory mediators, whereas A2AR antagonism reduced cytokine production under inflammatory conditions, further implicating A2AR in the modulation of microglial inflammatory polarization [11].

Furthermore, microglia-derived cytokines such as IL-1β, TNF-α, and IL-6 are widely recognized as major contributors to central sensitization and chronic pain pathophysiology. These mediators enhance excitatory neurotransmission, disrupt inhibitory signaling, and promote hyperexcitability of dorsal horn neurons, thereby facilitating amplification of nociceptive processing in chronic pain states [14–17]. In this context, the observed capacity of A2AR signaling to regulate microglial cytokine production suggests that A2AR-dependent inflammatory pathways may contribute to neuroimmune mechanisms implicated in pain sensitization, although nociceptive outcomes were not directly assessed in the experimental models described above.

### Astrocytic A2AR modulation and glial dysfunction

Beyond microglia, A2AR signaling has also been implicated in the regulation of astrocytic homeostasis and glial dysfunction under pathological conditions. Experimental evidence indicates that A2AR activation modulates multiple astrocyte-dependent processes, including glutamate transport, gliotransmitter release, and inflammatory reactivity [18, 19]. In particular, A2AR activation has been shown to inhibit astrocytic glutamate uptake and downregulate the glutamate transporters GLT-1 and GLAST through cAMP/PKA-dependent signaling, thereby favoring extracellular glutamate accumulation and excitatory imbalance [18, 19]. Mechanistically, this effect may involve direct interaction between A2AR and Na +/K + -ATPase α2, impairing the ionic gradient required for glutamate transporter function [18, 19].

In addition to neurotransmitter regulation, A2AR signaling also influences astrocytic intercellular communication. In APP/PS1 mouse models of Alzheimer’s disease, A2AR activity modulated astrocytic connexin 43 (Cx43) hemichannel dynamics and contributed to alterations in astrocyte morphology and signaling [20]. Pharmacological or genetic inhibition of A2AR prevented Aβ-induced dysregulation of Cx43 hemichannel activity, suggesting that A2AR contributes to astrocyte-mediated signaling disturbances under neurodegenerative stress [20, 21]. Complementary evidence indicates that A2AR-Cx43 interactions enhance ATP release from astrocytes, potentially establishing feed-forward purinergic signaling loops that further amplify glial activation [21].

Importantly, astrocyte dysfunction is increasingly recognized as a central contributor to chronic pain pathophysiology. Reactive astrocytes promote sustained neuroinflammation, modulate excitatory and inhibitory neurotransmission, and release cytokines and gliotransmitters that facilitate central sensitization and maladaptive synaptic plasticity within pain-processing circuits [27–29]. Therefore, the observed influence of A2AR signaling on astrocytic glutamate homeostasis, ATP release, and glial reactivity suggests that astrocytic A2AR modulation may represent an additional mechanism through which adenosinergic signaling contributes to pain-relevant neuroplastic and neuroinflammatory states.

### Inflammation-driven adenosinergic signaling and functional plasticity

Inflammatory states can substantially alter extracellular adenosine dynamics and thereby modulate A2AR-dependent neural signaling. Under conditions of tissue injury, hypoxia, or immune activation, stressed neural and glial cells release ATP into the extracellular space, which is subsequently converted into adenosine through the sequential action of ectonucleotidases such as CD39 and CD73 [22, 23]. Concurrently, inflammatory conditions may reduce adenosine reuptake and alter adenosine metabolism, further favoring extracellular adenosine accumulation and prolonged receptor activation [22, 23]. Together, these mechanisms establish an inflammatory microenvironment characterized by enhanced adenosinergic tone.

Elevated extracellular adenosine has been associated with functional impairment of adaptive neural plasticity. In a model of mild systemic inflammation induced by lipopolysaccharide (LPS), basal adenosine concentrations in the cervical spinal cord increased significantly, and this elevation was associated with impaired phrenic long-term facilitation (pLTF), a form of respiratory motor plasticity [24]. Pharmacological blockade of spinal A2AR restored pLTF under inflammatory conditions, supporting the notion that excessive A2AR activation contributes to inflammation-induced suppression of neural plasticity [24]. Similarly, in hippocampal inflammatory models, A2AR antagonism attenuated LPS-induced neuroinflammation and rescued long-term potentiation deficits, further implicating A2AR signaling in inflammation-mediated disruption of synaptic plasticity [24, 25].

In parallel, chronic metabolic inflammatory states may also engage this pathway. In diabetic rat models, increased hypothalamic A2AR expression has been associated with elevated TNF-α, oxidative stress markers, and autonomic dysfunction, further supporting a relationship between A2AR signaling and neuroinflammatory responses during persistent metabolic stress [26].

Notably, dysregulated neuroplasticity and inflammatory remodeling are widely recognized contributors to chronic pain pathophysiology. Neuroinflammation can alter excitatory and inhibitory circuit balance, promote maladaptive synaptic remodeling, and facilitate central sensitization across pain-processing pathways [30–32]. Therefore, the observed relationship between inflammation-induced adenosine accumulation, A2AR activation, and impaired neural plasticity suggests that this signaling axis may contribute to pain-relevant maladaptive plasticity, although direct nociceptive outcomes were not assessed in the experimental studies described above.

### Pharmacological and genetic modulation of A2AR-dependent pathways

Several studies demonstrate that pharmacological or genetic modulation of A2AR-associated pathways can significantly alter neuroinflammatory and neurofunctional outcomes across experimental models. Pharmacological blockade of A2AR has consistently been associated with attenuation of microglial activation, reduction of pro-inflammatory cytokine release, and mitigation of neuronal dysfunction in multiple neuroinflammatory contexts [33–35]. For example, selective A2AR antagonists have been shown to suppress inflammatory signaling and reduce oxidative and neurotoxic damage in glial and neuronal models of CNS injury [33]. Similarly, in BV2 microglia, knockdown of cystatin F reduced A2AR-mediated cytokine production, further supporting the role of downstream A2AR signaling networks in regulating inflammatory responses [13].

Functional improvements following A2AR inhibition have also been reported in models of inflammation-associated plasticity impairment. In LPS-treated animals, intrathecal administration of the A2AR antagonist MSX-3 restored phrenic long-term facilitation previously impaired under inflammatory conditions, suggesting that A2AR blockade may reverse inflammation-associated deficits in adaptive neural plasticity [24]. Comparable protective effects have been described in other neurological injury models, including traumatic brain injury, retinal injury, and Parkinsonian neuroinflammation, where A2AR antagonism reduced inflammatory burden and improved functional outcomes [35–37].

Importantly, some experimental studies have reported a more direct association between A2AR antagonism and pain-related outcomes. In preclinical pain models, selective A2AR antagonists have been associated with antinociceptive effects in experimental models, including reversal of mechanical allodynia in inflammatory pain paradigms, attenuation of glutamate-induced nociceptive behavior, and reduction of formalin-induced pain responses [10]. Furthermore, A2AR knockout mice exhibit hypoalgesic phenotypes and reduced spinal NMDA receptor binding, suggesting that A2AR signaling may influence nociceptive processing through both inflammatory and excitatory neurotransmission pathways [10, 38].

Collectively, these findings indicate that A2AR-dependent pathways may represent pharmacologically tractable targets for modulating neuroinflammation, neural plasticity, and pain-related signaling.

## Astrocytic A2ARs in synaptic regulation and glia–neuron communication

A2ARs act as fine modulators of astrocyte–neuron communication. Localized primarily to perisynaptic astroglial domains, these receptors regulate the amplitude and—most notably—the duration of intracellular Ca^2^⁺ responses and gliotransmitter release. Consequently, A2ARs shape the strength and temporal profile of astroglial signaling without serving as primary activation triggers. This modulatory function is mediated largely through cAMP/PKA-dependent mechanisms and does not necessarily require changes in purinergic receptor expression [39]. A2ARs further coordinate astroglial integration of P2X7 and P2Y1 receptor signaling, thereby organizing responses to extracellular “danger” signals and indirectly tuning synaptic activity and network organization [40].

In addition to regulating purinergic responsiveness, A2AR signaling has also been implicated in astrocytic control of glutamatergic homeostasis. Experimental evidence indicates that A2AR activation may impair astrocytic glutamate uptake through modulation of glutamate transport systems and associated ionic regulatory mechanisms, suggesting a broader role for A2AR in controlling extracellular excitatory balance [18, 19].

A2ARs also participate in receptor–receptor interactions with dopaminergic systems that contribute to context-dependent regulation of glia–neuron communication [41]. In the striatum, A2ARs and dopamine D2 receptors (D2Rs) are coexpressed in astroglial processes and form native heteromers in the plasma membrane. Functionally, A2AR–D2R heteromerization allows A2AR activation to antagonize D2R-mediated inhibitory control of astroglial glutamate release, thereby tuning gliotransmission and excitatory drive in a state-dependent manner [41]. Under aging and neurodegenerative conditions, disruption of this fine control contributes to synaptic homeostatic failure and impaired glia–neuron coordination [41–43].

Emerging evidence also suggests functional interactions between A2ARs and additional neuromodulatory systems, including oxytocinergic pathways, indicating that A2AR signaling may contribute to broader integration of neurochemical signals within astrocytic networks [39]. Collectively, these findings suggest a role for astrocytic A2ARs in regulating gliotransmission, synaptic homeostasis, and astrocyte-mediated modulation of neural circuit function. Although direct pain-related outcomes were not assessed in the reviewed studies, disruption of these astrocytic regulatory mechanisms may influence broader processes of network excitability and maladaptive plasticity implicated in chronic pain and other CNS disorders.

## A2A Receptor in neurological disorders with pain components

The role of A2AR in neurological and neurodegenerative disorders highlights its involvement in neuroimmune remodeling, particularly in conditions where chronic pain is frequently reported as a clinical feature. Under pathological conditions, dysregulated A2AR signaling may contribute to maladaptive plasticity and persistent neuroinflammation [24–26, 33–37, 42–46]. This dysfunction spans traumatic, metabolic, and demyelinating conditions, in which A2AR engagement has been associated with shifts toward a pro-inflammatory environment that may favor mechanisms implicated in neuropathic pain [42–46].

In traumatic brain injury (TBI), A2AR upregulation is linked to pathological hallmarks such as impaired perivascular aquaporin-4 (AQP4) polarization [42]. This astrocytic structural disruption compromises metabolic waste clearance and is associated with accumulation of hyperphosphorylated tau (p-tau) as well as significant remodeling of dendritic spine morphology [42]. Together, these alterations provide a mechanistic substrate for cognitive decline and broader neuronal dysfunction, processes that may indirectly contribute to altered sensory processing.

Demyelinating disorders further exemplify how aberrant A2AR signaling compromises neural integrity. A2AR activation exacerbates white matter injury, whereas pharmacological blockade reduces demyelinated lesion burden and improves spatial memory deficits [43]. Given that demyelination is frequently associated in central neuropathic pain, preservation of axonal insulation by A2AR antagonists suggests that receptor overactivation may contribute to mechanisms linking structural injury to persistent sensory dysfunction [43].

Metabolic stress provides an additional context in which A2AR-driven inflammation becomes clinically relevant. In diabetic complications, hyperglycemia-associated A2AR activation supports chronic low-grade neuroinflammation, characterized by microglial reactivity and vascular leakage [26, 44]. Targeting A2AR can restore vascular integrity and limit cytokine-driven inflammatory amplification, thereby potentially limiting neurodegenerative cascades associated with diabetic neuropathy and pain-related symptoms [44].

Glial activation has been widely implicated as an important component linking neural injury to persistent pain-related mechanisms, and A2AR signaling plays a prominent role in this transition. In microglia, A2AR activation promotes release of inflammatory mediators and engagement of complement pathways [12, 13, 33–36, 45]. This amplified glial inflammatory response may be further intensified by inflammasome activation (e.g., NLRP3 and caspase-1), contributing to cellular dysfunction and death [44, 45]. Consequently, sustained A2AR-driven neuroinflammation can hinder resolution of injury and foster chronic nociceptive signaling.

Emerging evidence suggests that neuropathic pain may involve broader dysfunction of the neural microenvironment rather than solely isolated sensory abnormalities [28, 29, 32]. In this context, A2AR-dependent shifts in glial phenotype may promote neuroimmune imbalance, maladaptive synaptic remodeling, and central sensitization associated with persistent pain states [14–17, 27–32, 42–45].

The therapeutic relevance of A2AR antagonism lies in its capacity to modify both disease progression and pain-related outcomes. By preventing dendritic spine loss and limiting toxic protein accumulation, A2AR blockade may target contributors to network dysfunction [10, 24, 33–38, 42]. This structural preservation may indirectly reduce pain by maintaining the integrity of sensory circuit architecture, supporting the premise that antagonist efficacy is strongly tied to restoration of neuroimmune homeostasis [42, 43].

The complexity of adenosine signaling is further highlighted by differential A2AR regulation across disease stages and compartments. While central A2AR overactivation is commonly deleterious, some conditions—such as idiopathic normal-pressure hydrocephalus (iNPH)—show downregulation of A2AR in peripheral cells [46]. This dissociation suggests that central A2AR antagonism may be neuroprotective, whereas systemic adenosine responses may vary across disorders, reinforcing the need for nuanced interpretation of A2AR signaling within global homeostasis [42, 46].

Notably, protective effects of A2AR modulation extend to specialized sensory systems. Microglial A2AR blockades prevent induction of pro-inflammatory mediators and complement components, thereby protecting neurons and photoreceptors from damage [45]. This ability to interrupt inflammatory persistence and cell death further supports A2AR as a broader regulator of neural survival and repair across multiple tissues [44, 45].

In summary, A2AR exerts context-dependent regulatory effects on neuroimmune remodeling, contributing to both physiological homeostasis and pathological dysfunction depending on the biological context. Under disease conditions, dysregulated A2AR signaling may promote glial activation, structural alterations, and persistent inflammation associated with neuropathic pain. Overall, targeted modulation of A2AR pathways may offer neuroprotective and therapeutic benefits by limiting neuroinflammatory and neurodegenerative processes relevant to pain pathophysiology.

## Affective dimension of pain: Involvement of the A2A receptor in behavior, mood, and pain perception

Although chronic pain has historically been framed as a predominantly sensory phenomenon, it is now widely recognized as a multidimensional experience integrating emotional, cognitive, and motivational components [1–3, 9, 10]. In view of the mechanisms discussed above—including persistent neuroinflammation, glial dysfunction, and A2AR-dependent modulation of synaptic signaling—adenosinergic pathways may influence neurobiological processes relevant to pain modulation, sensitization, and persistence [12–29, 39–41].

Accumulating evidence indicates that A2AR modulates circuits involved in mood, motivation, and stress responsiveness, particularly within prefrontal and limbic regions that also contribute to the affective appraisal of pain [46–48]. Alterations in adenosinergic signaling within these networks have been associated with sleep disturbances, shifts in arousal states, and changes in sensory processing—features commonly reported in chronic pain populations [46]. These observations align with broader mechanisms implicated in central sensization and suggest that A2AR dysfunction may influence processes associated with persistent sensory and emotional hypervigilance [14–17, 30–32, 46–48].

Furthermore, experimental and clinical studies reveal substantial overlap between chronic pain, anxiety, and depression—conditions that share partially convergent neurobiological and inflammatory mechanisms, including alterations in adenosinergic signiling [49, 50]. Within this context, A2AR may function as a relevant modulatory component linking immune activation, glial dysfunction, and behavioral disturbances. Sustained dysregulation of A2AR signaling may therefore contribute to mechanisms associated with nociceptive facilitation and the emotional burden frequently accompanying persistent pain states [12–21, 27–29, 33–37, 46–52].

From a translational perspective, A2AR antagonism has demonstrated beneficial effects in experimental models of behavioral dysfunction, including reductions in depressive- and anxiety-like phenotypes, normalization of sleep-related parameters, and improved motivation behavior [10, 33–38, 48]. Although these findings do not directly establish analgesic efficacy, modulation of emotional and cognitive domains may indirectly influence the broader context in which pain is experienced and managed [46–53].

Collectively, these findings reinforce the concept that chronic pain extends beyond nociceptive transmission and is shaped by broader neurobiological systems involved in affective and cognitive regulation. Within this framework, A2AR emerges as a potentially relevant integrative component linking neuroinflammatory activity, synaptic regulation, and behavioral processes. Further investigation is warranted to clarify the extent to which modulation of A2AR-dependent pathways may influence both nociceptive and affective dimensions of chronic pain [12–21, 27–32, 39–41, 46–52].

## Conclusion

Overall, this review highlights A2AR as an important regulatory node integrating neuroinflammation, glial reactivity, synaptic excitability, and affective circuitry, mechanisms implicated in chronic pain development and persistence. Across experimental and translational contexts, A2AR signaling has been associated with neuroimmune regulation, glial-neuroral communication and behavioral processes that may influence pain-related mechanisms and the emotional burden frequently accompanying persistent pain. Because these pathways overlap with mechanisms observed in neurological and psychiatric disorders frequently comorbid with chronic pain, A2AR may represent a promising therapeutic target beyond classical pain models. Future strategies aimed at A2AR modulation may therefore provide dual benefits by potentially attenuating central sensitization while also addressing affective dysregulation that perpetuates pain persistence.

## Data Availability

No datasets were generated or analysed during the current study.
